# Tolosa-Hunt syndrome mimicking cavernous sinus tumor

**DOI:** 10.5935/1808-8694.20130043

**Published:** 2015-11-02

**Authors:** Bernard Soccol Beraldin, Alexandre Felippu, Fabio Martinelli, Henrique Candeu Patricio

**Affiliations:** a Second-year Resident Physician at the Felippu Insttute of Otorhinolaryngology; b MD, ENT, Head Physician at the Felippu Insttute of Otorhinolaryngology; c MD, ENT, Preceptor at the Felippu Insttute of Otorhinolaryngology. Felippu Insttute of Otorhinolaryngology

**Keywords:** cavernous sinus, natural orifice endoscopic surgery, Tolosa-Hunt syndrome

## INTRODUCTION

The Tolosa-Hunt syndrome was first described in 1954, in the case report of a patient with painful ophthalmoplegia caused by nonspecific granulomatous inflammation of the cavernous sinus and the cavernous portion of the internal carotid artery. Seven years later, Hunt published a series of six patients and proposed the criteria to diagnose the syndrome, as follows: 1 - acute retro-orbital pain; 2 - alterations on the third, fourth, sixth, or first branch of the fifth cranial nerve and, less commonly, involvement of the optic nerve or sympathetic fibers around the cavernous portion of the carotid; 3 - symptoms persisting for days or weeks; 4 - spontaneous pain remission; 5 - recurrent episodes; 6 - prompt response to steroids^1^, ^2^.

Fifty-nine years after the description of this condition, the pathophysiological mechanisms and etiopathogenesis of the disease still remain controversial and obscure. This paper reports a case of Tolosa-Hunt syndrome in a patient first assumed to have a tumor in the cavernous sinus. Definitive diagnosis was obtained after a tumor was removed through endoscopic endonasal surgery.

## CASE REPORT

M.A, 60, female, reported onset of right-side retro-orbital pain and hemifacial paresthesia six years ago after undergoing dental treatment. She took painkillers to no avail, and was hospitalized as ordered by a neurologist. The patient was discharged three days later with no pain and mild residual paresthesia. Four years later the patient presented right-side hemifacial paresthesia. She was seen by two different ENTs and was diagnosed with and treated for sinusopathy and labyrinthopathy. Five months later she developed right-side diplopia, and was once again hospitalized by the same team of neurologists. A skull MRI was ordered and she was diagnosed with a probable tumor in the cavernous sinus.

Our team was then called to assess the patient, and we opted to attempt to remove the tumor through an endoscopic endonasal procedure. The transsphenoidal approach was used to reach the cavernous sinus. Scissors were used to open the anterior wall of the sphenoid sinus following a line parallel to the middle concha. The posterolateral bone wall was removed with chisel and hammer to expose the cavernous sinus. Four landmarks were then meticulously identified: 1 - the sphenoidal-orbital junction (anterior vertical boundary), 2 - the optic nerve (upper horizontal boundary), 3 - the maxillary branch (V2) of the trigeminal nerve (lower horizontal boundary), and 4 - the projection of the internal carotid artery (posterior vertical boundary). After the window was completed, the dura mater of the middle fossa was opened using three incisions: 1 - a horizontal incision, following a line below the projection of the optic nerve; 2 - a second horizontal incision, following a line above the projection of the maxillary nerve (V2); and 3 - a vertical incision, following a line in front of the most anterior projection of the internal carotid artery. The resulting flap was then lifted anteroposteriorly to reveal and allow access to the internal cavernous sinus.

**Figure 1:**
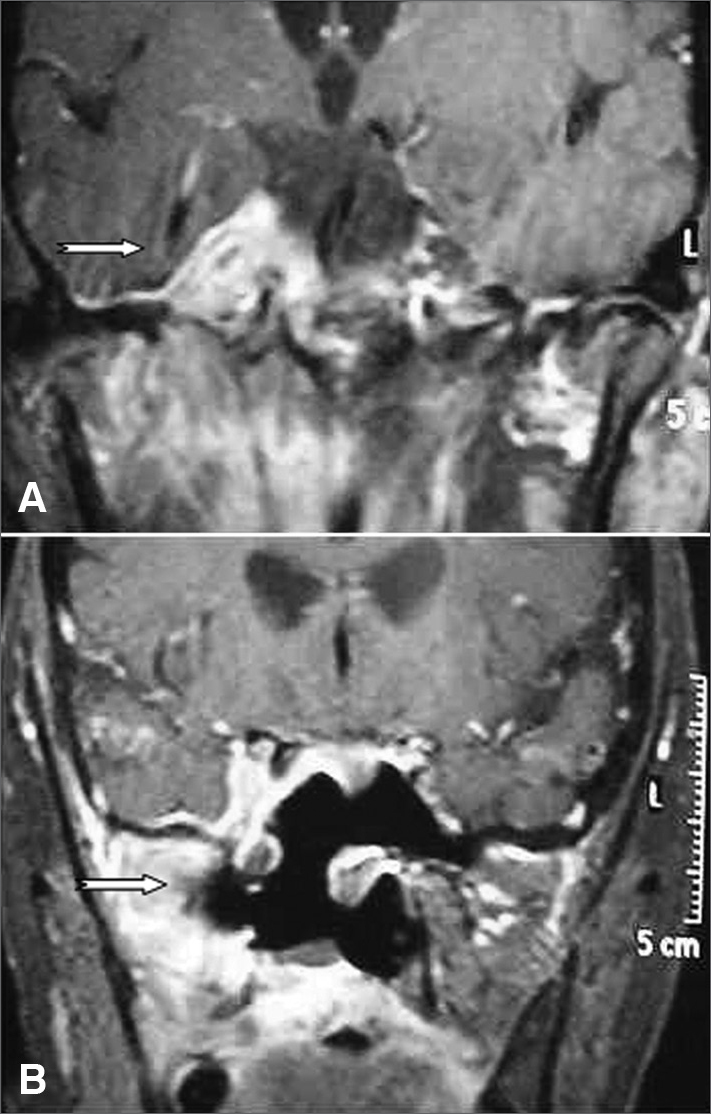
A: Preoperative skull MRI (contrast-enhanced T1). The arrow points to an expansive mass in the right cavernous sinus; B: Postoperative skull MRI (contrast-enhanced T1). The arrow points to the right cavernous sinus now without the mass.

The analysis of the specimen removed revealed nonspecific granulomatous inflammatory tissue. Immunohistochemistry assays (CD45, CD3, CD20, and CD30 to rule out lymphoma and other neoplasms) confirmed the diagnosis (Figure 1). Tolosa-Hunt syndrome was then considered and the patient was immediately put on high dose steroids. Two weeks into treatment the patient had improved significantly from pain, had no hemifacial paresthesia, and mild residual diplopia. The patient was asymptomatic after four weeks of treatment. Eight months after the end of drug therapy, the patient showed no signs of recurrence.

## DISCUSSION

The Tolosa-Hunt syndrome is a painful ophthalmoplegia characterized by unilateral orbital pain and oculomotor paresis that responds immediately to steroid therapy^3^. In 1988, the International Headache Society included the Tolosa-Hunt syndrome in the list of cranial neuralgias and, in 2004, the classification criteria for the syndrome were defined. Differential diagnosis includes diabetic neuropathy, cavernous sinus thrombophlebitis, ophthalmoplegic migraine, and tumors^4^, ^5^. Given the nonspecific nature of the disease's clinical findings, imaging and pathology testing can be used to accurately diagnose the syndrome.

Endoscopic surgery offers a safe and viable way to access the cavernous sinus when performed by properly trained personnel. The procedure does not require external approaches such as large craniotomies, and stands out as a less invasive and nearly atraumatic treatment option, in addition to shortening patient recovery time after surgery. The literature shows that treatment is based on clinical and radiological findings and does not include histology testing, a possibly valuable resource to that end.

## CLOSING REMARKS

The Tolosa-Hunt syndrome is a rare disease of unknown etiopathogenesis. The absence of a specific biologic marker for the syndrome requires the ruling out of all other causes of painful ophthalmoplegia, specifically when the patient is suspected for malignant disease, as in the case reported in this paper. Endoscopic endonasal surgery can be safely used to access the cavernous sinus and gather specimens for histology testing. Additionally, the procedure plays a fundamental role in the choice of treatment for patients with clinical or radiological signs of this disease.
